# T Follicular Helper-Like Cells in Inflamed Non-Lymphoid Tissues

**DOI:** 10.3389/fimmu.2018.01707

**Published:** 2018-07-23

**Authors:** Andreas Hutloff

**Affiliations:** Chronic Immune Reactions, German Rheumatism Research Centre Berlin (DRFZ), a Leibniz Institute, Berlin, Germany

**Keywords:** T follicular helper cells, B cells, inflamed tissue, germinal center, tertiary lymphoid structures

## Abstract

T and B cell cooperation normally takes place in secondary lymphoid organs (SLO). However, both cell types are also frequently found in inflamed non-lymphoid tissues. Under certain conditions, these infiltrates develop into ectopic lymphoid structures, also known as tertiary lymphoid tissues, which structurally and functionally fully resemble germinal centers (GCs) in SLO. However, tertiary lymphoid tissue is uncommon in most human autoimmune conditions; instead, relatively unstructured T and B cell infiltrates are found. Recent studies have demonstrated that active T and B cell cooperation can also take place in such unstructured aggregates. The infiltrating cells contain a population of T follicular helper (Tfh)-like cells (also designated “peripheral T helper cells”) lacking prototypic Tfh markers like CXCR5 and Bcl-6 but nevertheless expressing high levels of molecules important for B cell help like IL-21 and CD40L. Moreover, Tfh-like cells isolated from inflamed tissues can drive the differentiation of B cells into antibody-secreting cells *in vitro*. These findings are not restricted to experimental animal models but have been reproduced in rheumatoid arthritis and breast cancer patients. At this point, it is unclear whether T and B cell cooperation outside the ordered structure of the GC fully mirrors the reactions in SLO. However, Tfh-like cells in inflamed tissues are certainly important for the local differentiation of B cells into antibody-secreting cells, and should be considered as an important target for the treatment of autoimmune diseases.

## Introduction

The successful interaction of antigen-specific T and B cells is key to an effective humoral immune response resulting in the generation of high-affinity antibodies and long-term memory B cells. This interaction normally takes place in secondary lymphoid organs (SLO) where T and B cells interact in a temporally and spatially highly organized manner (1). The localization and movement of antigen-specific T and B cells is guided by their expression of chemokine receptors, which respond to defined chemokines locally produced by spatially restricted stromal cells. T follicular helper (Tfh) cells are the CD4^+^ T cell subset providing help for B cells (2). They are characterized by the transcription factor Bcl-6 and high expression of PD-1 and CXCR5, the latter chemokine receptor enabling their movement into the B cell zone. There, in the germinal center (GC) reaction, Tfh cells interact with antigen-specific B cells and drive their affinity maturation and further differentiation by means of the expression of CD40L and IL-21. As affinity maturation by somatic hypermutation always carries the risk of developing autoreactive B cell clones, the GC reaction has several safeguard mechanisms. First, GC B cells by far outnumber Tfh cells. Thereby, Tfh cell help is limited, which fosters selection of B cells with the highest affinity for specific antigen, since only these can efficiently present antigen to Tfh cells (3). This prevents expansion of poly-reactive B cells which also recognize self-antigens with low affinity. Second, the GC itself is further compartmentalized into a light zone, where antigen presentation and selection by Tfh cells occurs, and a dark zone, where B cells proliferate and hypermutate their B cell receptor. Cycling of B cells between these two micro-compartments and a rapid degradation of peptide–MHC complexes ensure that B cells are selected according to their actual B cell receptor affinity, and not an “old version” of the BCR retained on the cell surface (4). Finally, high-density presentation of the specific antigen by follicular dendritic cells (FDC) in the light zone provides B cells with an additional signal *via* their antigen receptor. Together, this stringent selection for highest antigen-affinity minimizes the risk of expanding B cells cross-reactive to auto-antigens.

Several recent reports have demonstrated that T and B cell interaction can not only take place outside of SLO but also even in structures completely lacking the highly organized microenvironment of the GC. Inflamed tissues may contain a unique population of non-classical Tfh cells, which can provide help for antigen-specific B cells. Here, I will review these findings and especially discuss their relevance for human autoimmune diseases.

## T and B Cells in Inflamed Tissues

Under chronic inflammatory conditions like autoimmune or allergic reactions, T and B cells are frequently found as infiltrates in non-lymphoid tissues. There, both cell types substantially contribute to tissue destruction by production of inflammatory cytokines. Since clonally expanded B cell populations in the inflamed tissue outnumber dendritic cells (5) and can efficiently take up low concentrations of antigen due to their high-affinity receptor, they play an important role as antigen-presenting cells and locally promote Th subset differentiation (6, 7). The other half of the interaction, the ability of T cells to provide B cell help, and signals for local B cell differentiation is frequently neglected, although it too contributes to pathology.

## Ectopic Lymphoid Structures (ELS)

Under certain conditions, T and B cell infiltrates in inflamed tissues develop into ELS, also known as tertiary lymphoid tissue (8, 9). These structures anatomically and functionally fully resemble SLO; they are characterized by separated T and B cell zones, the presence of FDC, and high endothelial venules, which enable T and B cells to enter these structures. Within ELS classical GC reactions take place with the presence of CXCR5^+^ Bcl-6^+^ Tfh cells and GC B cells highly proliferating and expressing the cytidine deaminase AID, which is the key enzyme for somatic hypermutation and immunoglobulin class switching. In a mouse model lacking all SLO it was shown that ELS can fully replace their function (10). In human autoimmune diseases, they are considered to play an important role in somatic hypermutation of autoreactive B cells and plasmablast generation directly in the affected tissues (8, 9).

While research on T and B cells in inflamed tissues primarily focused on these ELS, it also became clear that their development requires rather strong stimuli. This was nicely demonstrated in a mouse model where a lung infection with vaccinia virus was directly compared to a bacterial infection with *Pseudomonas aeruginosa* (11). In both cases, prominent lymphoid infiltrates developed in the lung. However, only in the viral infection model did fully developed, FDC-positive ELS evolve.

Moreover, in human autoimmune diseases, only a fraction of lymphoid infiltrates in inflamed tissues are characterized by fully developed ELS (Table 1). In rheumatoid arthritis patients, where ELS were first described in the synovial membrane of inflamed joints, early studies reported an incidence of fully developed, FDC-containing, and thereby GC-like infiltrates of approximately 25% (12–14). The remaining samples contained either mainly T cells diffusely distributed over the whole tissue or clusters of T and B cells lacking segregation into T and B cell zones and not containing any FDC. However, two more recent studies analyzing larger numbers of samples and more specific parameters came to the conclusion that fully developed, FDC-positive ELS are rather rare in synovial tissue from arthritis patients with a prevalence of only 6–8% (15, 16). This of course might also be related to substantially improved patient treatment regimens in the past years resulting in fewer cases with severely inflamed, end-stage joints. Importantly, patients with fully developed FDC^+^ ELS did not differ from patients with unstructured T and B cell infiltrates regarding several clinical parameters including positivity for rheumatoid factor and anti-citrullinated protein antibodies, suggesting that similar disease processes are occuring in patients with or without FDC^+^ ELS (15, 16).

**Table 1:** Prevalence of fully developed ectopic lymphoid structures (ELS) in human autoimmune diseases.

Disease	Affected organ	Prevalence of ELS^a^ (%)	Reference^b^
Rheumatoid arthrits	Synovial joint	6–35	(13–17)
Rheumatoid arthrits	Lung	11	(18)
Systemic lupus erythematosus	Kidney	6	(19)
Sjögren’s syndrome	Salivary glands	10–43	(20–23)
Myositis (adult)	Muscle	0	(24)

*^a^Follicular dendritic cells-positive or with clearly segregated T and B cell zones*.

*^b^Considering only studies with >15 patients*.

Also for other autoimmune conditions like systemic lupus erythematosus (SLE), more recent studies revised earlier reports of highly incident fully developed ELS and demonstrated the predominant presence of unstructured, FDC-negative T and B cell aggregates (19). In adult autoimmune myositis, fully developed ELS are completely absent. Nevertheless, B cell receptor sequence analysis from single muscle infiltrates revealed clonally related sequences and locally ongoing somatic hypermutation (24).

## Tfh-Like Cells in Inflamed Tissues

In view of the fact that fully developed ELS are rather an exceptional than a common finding in human autoimmune diseases, several recent reports demonstrating that active T and B cell cooperation can also take place in inflamed tissues in unstructured, FDC-negative infiltrates are of high interest. The first indication that highly activated ICOS^+^ CD4^+^ T cells interact with B cells in FDC-negative, unstructured infiltrates and might drive their local differentiation into plasmablasts came from a study analyzing kidney infiltrates in SLE patients (25). In 2008, a Tfh-like but CXCR5- and Bcl-6-negative T cell population was described in the synovial joint of rheumatoid arthritis patients (26). These cells produced the human Tfh signature chemokine CXCL13 and even higher amounts of IL-21 than classical Tfh cells from human tonsil. They were mainly located outside of FDC^+^ GC-like structures and even found in diffuse infiltrates (26, 27). Similar cells could be induced *in vitro* under the influence of TGF-β (28). Only recently, Tfh-like cells from the synovium were characterized in more detail and found to be distinguished by very high expression of PD-1 and chemokine receptors directing migration to inflamed tissues like CCR2 and CCR5 (29). Moreover, it was demonstrated in an *in vitro* T and B cell cooperation assay that these Tfh-like cells can indeed provide B cell help to differentiate B cells into antibody-secreting plasmablasts. Also in the inflamed kidney of lupus nephritis patients, a Tfh-like population expressing high levels of PD-1, ICOS, and CXCR4 was recently described (30). Like in arthritis patients, these cells produced large amounts of IL-21 and were located outside of GC-like structures, but in close contact with B cells.

Similar Tfh-like cells were recently also found in human breast cancer, where their presence was associated with a positive prognosis for the patients (31, 32). These cells lacked the classical Tfh cell-defining markers CXCR5 and Bcl-6 but were positive for other typical markers like PD-1, ICOS, and TIGIT. Furthermore, they were characterized by high expression of IL-21 and CXCL13. Importantly, these cells were not only present in FDC^+^ areas but also in lymphocytic infiltrates near the tumor bed in close contact with tumor-infiltrating B cells.

A more detailed functional analysis of this novel subset of tissue-infiltrating Tfh-like cells became possible by the use of two different lung inflammation mouse models (33, 34). In a house dust mite-induced asthma model, a distinct population of IL-21-producing CD4^+^ T cells was identified in the inflamed lung, where these cells most likely contributed to airway eosinophilia and allergen-specific IgG1 production. These cells expressed high levels of PD-1 but lacked expression of CXCR5 and were also not of Th2 or Th17 lineage origin. In a second lung inflammation mouse model, a similar Tfh-like population was found in lung infiltrates in close contact with GC-like B cells, however, these infiltrates completely lacked any structured GC-like properties (34). High proliferation of lung-resident B cells and the ability of isolated lung T cells to drive differentiation of naive B cells into IgA^+^ plasmablasts *in vitro* demonstrated their B helper potential. These Tfh-like cells were CXCR5- and Bcl-6-negative but expressed even higher levels of PD-1, ICOS, CD40L, and IL-21 than classical Tfh cells in the lung-draining lymph node (34). Apart from the lack of CXCR5 and Bcl-6 expression, their unique characteristics as a cell lineage distinct from classical Tfh cells was demonstrated by the finding that their high expression of PD-1 was independent of ICOS costimulation (5).

Very recently, another Tfh-like cell population was described in a mouse model for systemic sclerosis (35). These PD-1^high^ ICOS^high^ T cells in the skin contributed to fibrosis by their strong production of IL-21. Although these cells were negative for Bcl-6, they expressed significant levels of CXCR5, which clearly discriminates them from the Tfh-like cell populations described above.

## GC-Like Reactions in Inflamed Tissues

These novel findings that a population of Tfh-like cells can provide help to B cells in inflamed tissues in the absence of any ordered GC-like structures of course raise the question, how these T/B interactions can take place, and which consequences arise for the selection of antigen-specific plasma cells and memory B cells.

The presence of FDC has been always considered as a hallmark of the GC. However, as shown in the above-mentioned lung inflammation mouse model, B cells with a phenotype similar to classical GC B cells in SLO (PNA^+^ GL-7^+^ CD38^low^ Bcl-6^+^ AID^+^) can also develop in unstructured, FDC-negative infiltrates in non-lymphoid tissues (34). In this regard, two earlier studies with lymphotoxin β-deficient mice, which lack FDC in all lymphoid organs, are of special interest. Nevertheless, upon immunization these mice developed phenotypically and functionally normal GC (36, 37). Only affinity maturation of the B cell receptor was reported to be delayed in one study (36), whereas the second study demonstrated a shortened persistence of the GC (37). Moreover, in an autoimmune mouse model, B cells undergoing active somatic hypermutation were found outside of FDC-positive B cell follicles (38). Together this shows that other cell types can take over the antigen-presenting function of FDC. In addition to other activated B cells, non-FDC stromal cell populations and macrophages, which are an integral part of leucocytic infiltrates in inflamed tissues, might be good candidates (11, 39).

A second key function of the FDC network in SLO is the spatial organization of the GC. By production of CXCL13, FDC attract both CXCR5^+^ Tfh cells and B cells into the same compartment. Whereas FDC are the main producers of CXCL13 in SLO, other cell types have been shown to take over this function in inflamed tissues (39). At least in the human, the Tfh-like cells in the infiltrates are also a source of CXCL13 (26, 27, 29, 31). However, as Tfh-like cells lack expression of the chemokine receptor CXCR5, recruitment *via* CXCL13 is not an option for the T cells but might be a possibility for B cells, which still express CXCR5 in inflamed tissues, albeit at a reduced level compared to SLO (40). Thereby, other chemokines must be responsible for the striking clusters of T and B cells observed in defined areas of the inflamed tissue. This could be CXCL12, which was shown to be produced by a podoplanin-positive stromal cell population in FDC-negative T and B cell clusters in the inflamed lung (11). Both B cells and Tfh cells express the corresponding chemokine receptor CXCR4. Another good candidate for T and B cell recruitment into inflamed tissues is CXCR3, which is expressed on T and B cells under inflammatory conditions (40, 41). Moreover, very high levels of the corresponding chemokine CXCL10 have been found in the inflamed synovium of rheumatoid arthritis patients (42).

Although recruitment of antigen-specific T and B cells into spatially defined areas of the inflamed tissue can clearly occur without FDC, a highly organized structural segregation into defined T and B cell zones as in SLO is absent (17, 34). On one hand, this facilitates optimal T cell/B cell contact, especially since T cells are not limited as in the classical GC but typically even outnumber B cells. On the other hand, this permanent availability of T cell help, by increasing the chances of T:B interaction, will decrease the relative competition among B cells for high-affinity antigen. Since the stringent selection for highest antigen affinity like in the classical GC is missing, low-affinity cross- or poly-reactive B cells—including autoreactive B cells—easily become selected.

Another hallmark of GC reactions in SLO is the generation of long-lived plasma cells and memory B cells (1). Whether the plasmablasts numerously found in inflamed tissues later migrate to the bone marrow to become long-lived plasma cells is not known and also difficult to determine experimentally, since the T/B interactions in inflamed tissue are always accompanied by a classical GC reaction in the tissue-draining lymph node. However, it has been clearly demonstrated that antigen-specific Tfh-like cells and B cells generated in the lung tissue could survive locally and in the absence of antigen as long-term tissue-resident memory cells (34). In the same model, memory T and B cells from the immune reaction in the lung were also found as circulating memory cells in spleen and blood. At the moment, it is not clear whether these were cells originally generated in the lung-draining lymph node or whether lung-resident T and B cells have the ability to migrate to other tissues. This is an important question, since it has been discussed for rheumatoid arthritis patients whether autoreactive T and B cells originally generated in the lung might later migrate to joints to induce synovial inflammation (43).

## Extrafollicular Tfh Cells

Another unconventional Tfh cell subset are so called extrafollicular Tfh cells. They were first identified in the spleens of a lupus mouse model as CD4^+^ PSGL-1^low^ CXCR5^low^ CXCR4^+^ T cells producing large amounts of IL-21 and CD40L (44). In contrast to the tissue-resident Tfh-like cells described above, they clearly belong to the Tfh cell lineage as they express Bcl-6 and require it for their development (45). Due to their very low expression of CXCR5, extrafollicular Tfh cells are not located in the GC but at the T/B border where they drive differentiation of extrafollicular plasmablasts (44). In lupus-prone mice, they are not only found in SLO but also the inflamed kidney where they substantially contribute to pathogenesis (46–48).

## Conclusion

Recent studies have shown that the ability of B cell help is not exclusive to classical Bcl-6^+^ CXCR5^+^ Tfh cells. Instead, a phenotypically distinct but functionally similar Tfh-like subset can take over this function, especially in inflamed tissues. This is an important notion also in regard to therapeutic strategies to eliminate Tfh cells in chronic inflammatory conditions. Due to their distinct gene expression profile, Tfh-like cells might be refractory to therapies targeting classical Tfh cells like B cell depletion or ICOS costimulation blockade. At the same time, tissue-resident Tfh-like cells are probably the most pathogenic T cell subset, as they select autoreactive B cells in the uncontrolled environment of lymphocytic tissue infiltrates and drive the local differentiation of plasmablasts producing pathogenic antibodies directly in the affected tissues.

## Author Contributions

The author confirms being the sole contributor of this work and approved it for publication.

## Conflict of Interest Statement

The author declares that the research was conducted in the absence of any commercial or financial relationships that could be construed as a potential conflict of interest.
